# The Tolerability and Efficacy of 4 mA Transcranial Direct Current Stimulation on Leg Muscle Fatigability

**DOI:** 10.3390/brainsci10010012

**Published:** 2019-12-23

**Authors:** Craig D. Workman, John Kamholz, Thorsten Rudroff

**Affiliations:** 1 Department of Health and Human Physiology, University of Iowa, Iowa City, IA 52242, USA; Craig-workman@uiowa.edu; 2 Department of Neurology, University of Iowa Hospitals and Clinics, Iowa City, IA 52242, USA; john-kamholz@uiowa.edu

**Keywords:** transcranial direct current stimulation, muscle fatigue, high intensity, tolerability

## Abstract

Transcranial direct current stimulation (tDCS) modulates cortical excitability and affects a variety of outcomes. tDCS at intensities ≤2 mA is well-tolerated, but the tolerability and efficacy of tDCS at intensities >2 mA merits systematic investigation. The study objective was to determine the tolerability and effects of 4 mA tDCS on leg muscle fatigability. Thirty-one young, healthy adults underwent two randomly ordered tDCS conditions (sham, 4 mA) applied before and during an isokinetic fatigue test of the knee extensors and flexors. Subjects reported the severity of the sensations felt from tDCS. Primary outcomes were sensation tolerability and the fatigue index of the knee extensors and flexors. A repeated-measures ANOVA determined statistical significance (*p* < 0.05). Sensation severity at 4 mA tDCS was not substantially different than sham. However, two subjects reported a moderate–severe headache, which dissipated soon after the stimulation ended. The left knee flexors had significantly greater fatigability with 4 mA tDCS compared with sham (*p* = 0.018). tDCS at 4 mA was well-tolerated by young, healthy subjects and increased left knee flexor fatigability. Exploration of higher intensity tDCS (>2 mA) to determine the potential benefits of increasing intensity, especially in clinical populations with decreased brain activity/excitability, is warranted.

## 1. Introduction

Transcranial direct current stimulation (tDCS) is a non-invasive brain stimulation technique [1,2,3,4] that can modulate the cortical excitability of targeted brain regions [5]. tDCS has been extensively tested in healthy subjects [6,7,8] and in people with cognitive [7] and motor impairments [9,10,11], and these studies suggest that tDCS can help improve cognitive and motor function. However, an important caveat is that the majority (~96%) of tDCS sessions to date have been limited to 2 mA or lower [12]. This standard of 2 mA has become an unofficial ‘upper limit’ for tDCS research and is likely founded from safety concerns of an historical case in which subjects suffered mutism, quietness, and nausea from high-dose stimulation (e.g., ~1.27 cm electrodes; stimulation durations >1 h) [13]. Thus, most have selected more conservative stimulation parameters and it remains unclear how higher intensity tDCS (i.e., >2 mA) affects cognitive and motor function. However, given the improvements of tDCS technologies and methodologies, exploration of higher intensities is not only plausible, but recommended [14].

The tolerability of tDCS has been investigated and compared for intensities ≤2 mA. For example, one study reported increasing discomfort with increasing intensity from 0.75 mA to 2 mA [15], while another found no significant discomfort from graded intensities ranging from 1 mA–4 mA [16]. Furthermore, there is empirical evidence that 2 mA tDCS for 20–40 min, in either single or multiple sessions, can be safely administered to healthy subjects [12]. Additionally, data from more than 33,200 sessions and 1000 subjects that experienced repeated sessions, including those from potentially vulnerable populations, have yielded no reports of serious adverse effects or permanent injuries when using conventional tDCS protocols (≤40 min, ≤4 mA, ≤7.2 Coulombs) [12].

The tolerability of tDCS at intensities >2 mA is a meaningful issue to explore because higher intensity stimulation might further increase cortical excitability, and thus increase motor and cognitive improvements, in healthy and clinical populations. However, the assumption that a higher intensity enhances clinical outcomes in a safe and tolerable manner has not been systematically explored. There are a few that have used intensities >2 mA and have found that 4 mA was safe and well tolerated in stroke survivors [14,16], patients with movement disorders [17], and patients with major depression [18], but each had a very small number of subjects (*n* < 3). Additionally, a recent report testing an adaptive stimulation controller found that 4 mA tDCS was well tolerated by young adults [19]; however, they did not explore the effects of higher intensity stimulation on a motor task. Thus, investigation of the effects and tolerability of intensities >2 mA is still necessary.

Furthermore, the timing of tDCS, and the effect on tolerability, is still ambiguous. No tDCS study has investigated the effects and tolerability of a 4 mA intensity during a motor or cognitive task. Theoretically, tDCS applied during a motor task (e.g., a fatigue task) could lead to enhanced performances compared with tDCS applied before the same task. This is because tDCS of the resting motor cortex may excite neuronal populations in a non-specific way (i.e., not in the same way as when actually performing the motor task) [20]. In contrast, tDCS applied during a task may further enhance the normal increases in cortical excitability and synaptic efficiency in specific, task-related cortical circuits active during task execution. This enhancement could lead to an improved performance and potentially enhanced plasticity. Furthermore, administering tDCS during a task may improve subjective tolerability as the task itself may serve as a distractor to the potentially bothersome stimulation.

Fatigue is an important clinical outcome in tDCS studies and has been investigated in healthy subjects [21,22,23,24] and patients with neurological or psychiatric disorders [25,26,27,28]. However, tDCS studies on fatigue, especially in neurological disorders, have elicited conflicting results. For example, Proessl et al. [28] showed no effect of 2 mA tDCS, using a unilateral montage (anode: more affected M1; cathode: contralateral supraorbit) on time to task failure. They applied tDCS 90 s before and then during an isometric fatigue task of the more-affected leg extensors in people with Multiple Sclerosis (PwMS). Other studies in PwMS also showed no significant improvements on fatigue questionnaires from unilateral prefrontal or parietal tDCS stimulation applied daily for five consecutive days [29,30,31]. However, tDCS applied daily for five consecutive days over the primary somatosensory cortex (S1) bilaterally [32,33,34], the primary motor cortex (M1) bilaterally [35], and the sensorimotor cortex bilaterally [33], showed significant fatigue improvements. The discrepancy in the results might be related to the differences in stimulation targets, protocol duration (single session vs. five daily sessions) and the adapted current intensity applied in these studies (e.g., all of these protocols used intensities ≤2 mA).

Although tDCS is ultimately aimed at improving the quality of life of clinical populations, it is important to first explore the tolerability and efficacy of higher intensity tDCS (>2 mA) in healthy individuals. Therefore, the purpose of this study was to investigate the effects of 4 mA tDCS applied before and during a fatigue task on stimulation tolerability and leg muscle fatigability in healthy subjects. It was hypothesized that the 4 mA condition would be well-tolerated by the subjects and that the 4 mA condition would yield altered fatigue profiles compared to the sham condition.

## 2. Materials and Methods

### 2.1. Subjects

Thirty-four physically active, right-side dominant young adults were recruited (females = 22; mean ± SD, age = 24 ± 3.6 years, height = 169.2 ± 9.9 cm, weight = 71.2 ± 13.3 kg). Inclusion criteria included: (1) Young adult (18–30 yrs.); (2) right-side dominant; (3) ≥30 min of moderate intensity physical activity ≥3 days per week for ≥3 months; (4) without chronic neurological, psychiatric, or medical conditions; and (5) not taking any psychoactive medications. The exclusion criteria included: (1) Pregnant; (2) known holes or fissures in the skull; (3) metallic objects or implanted devices in the skull (e.g., metal plate). This study was approved by the University of Iowa’s Institutional Review Board and all subjects provided written informed consent prior to participation.

### 2.2. Experimental Protocol

This study employed a double-blind, randomized, sham-controlled, cross-over design. Subjects completed three visits to the laboratory, each separated by 5–8 days. During the first visit, right-side dominance was verified via an isokinetic maximal strength test of both legs and the subjects were familiarized with the fatigue test (see subsections below for test details). Right-side dominant subjects were recruited to avoid the potential for different functional brain morphology between right-and left-dominant people [36]. After completing the strength testing, an isokinetic fatigue test (FT) was performed on each leg, with <1 min between ending the right leg FT (R-FT) and starting the left leg FT (L-FT). For consistency between subjects and tDCS sessions, strength and fatigue testing was always performed on the right leg first. During visits two and three, one of two randomly ordered tDCS sessions (sham and 4 mA; details below) were administered and R-FT and L-FT were repeated.

### 2.3. Isokinetic Strength Test

Strength and fatigue testing were performed on an isokinetic dynamometer (HUMAC NORM, CSMi, Stoughton, MA, USA). The strength test started with a 15 repetition submaximal warm-up of the knee extensors and flexors (concentric/concentric, 60°/s), followed by five sets of one maximal effort knee extension and flexion (concentric/concentric, 60°/s [37]), with ≥30 s rest between sets. The largest torque was retained for dominance verification (i.e., right stronger than left). Verbal encouragement was provided to help the subjects achieve a maximal effort.

### 2.4. Isokinetic Fatigue Test

The FT consisted of 40 continuous maximal concentric/concentric contractions of the knee extensors and flexors performed at 120°/s [38]. On visits two and three, the FT was preceded by the same 15 repetition warm-up detailed above. Once the warm-up was completed, and at the appropriate time during tDCS (see Section 2.5), the R-FT was completed followed immediately by the L-FT. Total time to complete both FTs (including transitioning between right and left sides) was 4.5–5 min. Again, verbal encouragement was provided to help the subjects achieve a maximal effort for each repetition.

### 2.5. tDCS Sessions

**Figure 1:**
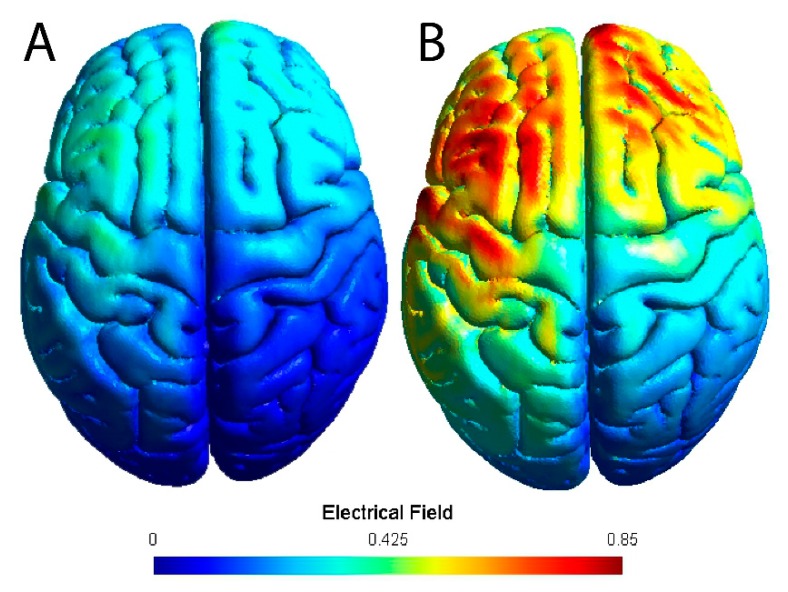
Model of the electrical field of the transcranial direct current stimulation montage used. The anode was placed over C3 and the cathode over the contralateral supraorbital area. (**A**) Electrical field using a standard 2 mA intensity. (**B**) Electrical field using a 4 mA intensity.

Stimulation was delivered with a battery powered 1 × 1 tDCS Low-Intensity Stimulator (Model 1300A, Soterix Medical Inc., New York, NY, USA) using two carbon electrodes placed inside 5 × 7 cm (35 cm^2^ surface area; current density = 0.11 mA/cm^2^) EASYpad sponges (Soterix Medical Inc., New York, NY, USA) soaked with 10–15 mL of 0.9% NaCL saline. The sponges were held in place using an EASYstrap (Soterix Medical Inc., New York, NY). The anode was placed over the left M1 (i.e., C3 using the 10–20 EEG placement convention [39]) and the cathode was over the contralateral supraorbital area. Figure 1 shows a model of the theoretical electrical field of this montage for a standard 2 mA intensity and the higher 4 mA intensity. The dominant (left) M1 was targeted to maximize motor performance [40]. The 4 mA tDCS condition started with a 30 s ramp-up to 4 mA, after which it remained at 4 mA for 20 min, followed by a 30 s ramp-down to 0 mA. The sham condition included the 30 s ramp-up to 4 mA and a 30 s ramp-down to 0 mA at the beginning and the end of the stimulation time (20 min). In the intervening time, the intensity was set to 0 mA.

The stimulation device included a ‘PRE-STIM TICKLE’ function that activated a 1 mA stimulation for ~30 s. This was used to test electrode contact quality and to help determine if the electrodes had sufficient skin/scalp contact; readjustments to the electrode contact and sponge saturation were made as necessary. The PRE-STIM TICKLE was performed prior to starting tDCS to ensure high quality contact. Furthermore, to ensure consistency between tDCS sessions, the location of each electrode on the EASYstrap, which has distance (cm) markings similar to a ruler, was recorded at the second visit and the sponges were placed in the same position at the third visit. Both sham and 4 mA tDCS were administered for a total of 20 min with the subject seated in the dynamometer chair. However, at minute 15, the subject started the R-FT, followed immediately by L-FT. Thus, sham or 4 mA tDCS was administered before and during the FT of both legs. The same researcher administered tDCS to all subjects and knew the stimulation condition, but the rest of the test administrators and subjects were blinded to the stimulation order (sham and 4 mA).

To determine the tolerability of tDCS at 4 mA, the subjects were asked to describe any sensations felt during stimulation (e.g., itching, tingling, burning, etc. [41]) and to rate the severity of those sensations on a 10-point Likert-type scale (1 = lowest, 10 = highest) at the end of each tDCS condition. Additionally, the subjects were asked to guess which tDCS condition they experienced at the end of each session. Feedback was not provided until all tDCS sessions and sensation descriptions were completed.

### 2.6. Data Analysis

**Figure 2:**
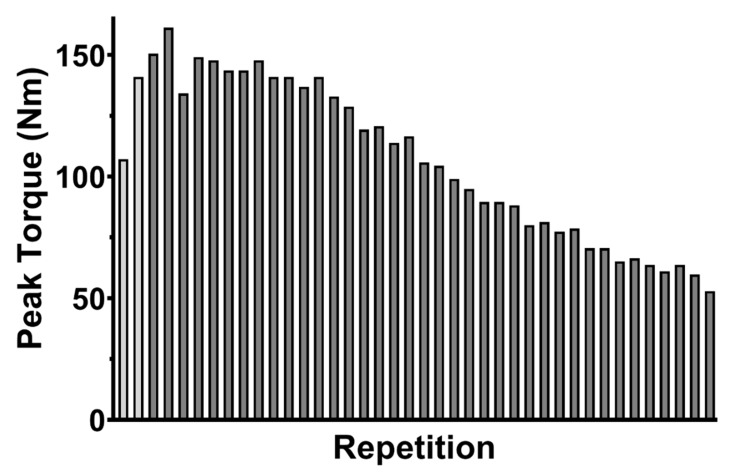
Peak torque of the right knee extensor muscle group during a fatigue test for a representative subject. The bars represent the maximum torque achieved during the repetition. Note that the first two (light gray) were considered adaptation repetitions and were not included in the fatigue index calculation.

Tolerability of tDCS was determined by averaging the severity of reported sensations and blinding was assessed by calculating the percent of correct tDCS condition guesses. The primary outcome for the FT was the fatigue index (FI; percent decline in torque production from the beginning to the end of the FT) for the right and left knee extensors and flexors. The fatigue index was calculated as follows: FI = ([mean of the first five repetitions—Mean of the last five repetitions]/mean of the first five repetitions) * 100 [42,43,44]. To control for any adaptation effects at the beginning of the FT, the first two repetitions of each FT were not included in the FI calculation (Figure 2). Thus, the mean of the first five repetitions in the FI calculation represented the mean of repetitions three to seven.

### 2.7. Statistical Analysis

A 2-factor repeated-measures ANOVA, stimulation (sham vs. 4 mA) by knee muscle group (right extensors vs. left extensors vs. right flexors vs. left flexors), was performed to analyze the FI data. Additionally, to control for potential bias in the FI data from less-motivated subjects, FI results ≤0% (i.e., those with higher torque production at the end of the trial) were removed from statistical analysis. Due to the exploratory nature of the analysis, uncorrected post-hoc pairwise comparisons (paired *t*-tests) and effect sizes (Cohen’s d) were performed to elucidate significant tDCS differences. Significance was accepted at *p* < 0.05 and analysis was performed using SPSS 25 (IBM Corp, Armonk, NY, USA).

## 3. Results

Of the 34 subjects recruited, two could not endure the PRE-STIM TICKLE contact quality check at the beginning of the second visit and withdrew from the study, and one attended the baseline session but did not keep any remaining appointments and was lost to contact. The remaining 31 completed all scheduled sessions. Of these, four had FI results that did not meet the FI bias-correction cut-off (FI ≤ 0%), and their data were not included in the FI statistical analysis (total *n* = 27); however, the tolerability and blinding reports of these subjects were still included (total *n* = 31).

The tolerability and blinding results are displayed in Table 1. In general, the sham and 4 mA tDCS were equally well-tolerated. The 4 mA tDCS condition had a slight increase in the number of subjects reporting a few sensations (e.g., burning and itching), accompanied by a slight increase in sensation severity, which was still considered mild. Additionally, a few new sensations were reported in the 4 mA condition. Most notable in Table 1 is the report of a moderate–severe headache by two subjects. Nevertheless, these subjects were still able to complete the 20 min of stimulation and the FT. All subjects reported that the sensations felt during either sham or 4 mA tDCS dissipated within minutes of turning off the tDCS device, including the two that reported a headache.

**Figure 3:**
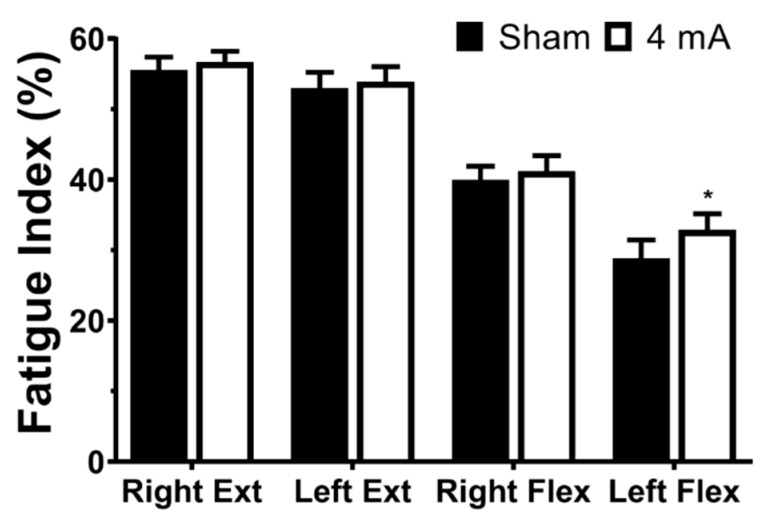
Significant effects of transcranial direct current stimulation (tDCS) on fatigue index. Data are mean ± SEM. Ext = knee extensor muscle group, Flex = knee flexor muscle group. * indicates sham significantly different than 4 mA (*p* = 0.018).

The results of the repeated-measures ANOVA on the FI data indicated a significant effect of muscle group (*p* < 0.001) and stimulation (*p* = 0.022), but not a stimulation × muscle group interaction (*p* = 0.347). The pairwise testing revealed that, for both the right and left legs, the knee extensors had a significantly larger FI percentage (i.e., greater decrease in torque production from the beginning to the end of the FT) than the knee flexors in both sham and 4 mA tDCS conditions (all *p* < 0.001). Additionally, the right knee flexors had a significantly larger FI percentage than the left knee flexors in both tDCS conditions (*p* < 0.001). For the stimulation effects, post-hoc testing indicated that 4 mA tDCS had a larger FI than sham for the left knee flexors (mean ± SD: sham = 28.8 ± 13.6 vs. 4 mA = 32.9 ± 11.9, *p* = 0.018, d = 0.32; Figure 3). There were no stimulation-related changes for the right knee extensors (sham = 55.5 ± 9.6 vs. 4 mA = 56.7 ± 8.0, *p* = 0.378, d = 0.13), the left knee extensors (sham = 53.0 ± 11.8 vs. 4 mA = 53.9 ± 11.0, *p* = 0.477, d = 0.08), or the right knee flexors (sham = 40.0 ± 10.0 vs. 4 mA = 41.2 ± 11.4, *p* = 0.423, d = 0.11).

## 4. Discussion

The purpose of this study was to investigate the tolerability of 4 mA tDCS stimulation and to determine the effects of 4 mA tDCS on leg muscle fatigability. The novel findings were that 4 mA tDCS was well-tolerated and that 4 mA tDCS increased the fatigability of the left knee flexors of young, healthy subjects.

The tolerability reports from the subjects indicated a favorable profile for 4 mA tDCS, with the frequency and intensity of sensations very similar to sham (Table 1). These tolerability reports are similar to previous reports using tDCS ≤ 2 mA [1,15], but differ from another in which only a few people with stroke (maximum n for any intensity = 2) reported skin redness (and no other sensations) from tDCS using intensities 1 mA–4 mA for 30 min [16]. Importantly, the tolerability profile presented in Table 1 in [16] only included sensations experienced after, but not during, tDCS; in the present study, subjects reported sensations felt at any time during stimulation. However, the authors in [16] did mention that transient sensations similar to those reported here were felt early in the stimulation period, and were always mild.

Of note, two subjects in this study experienced relatively severe headaches in the 4 mA condition, which quickly resolved after stimulation ended. Additionally, it is important to emphasize that the two subjects that withdrew for stimulation-related reasons withdrew before experiencing 4 mA tDCS. As mentioned above, these subjects could not endure the PRE-STIM TICKLE contact quality check, which had a maximum intensity of 1 mA. For these participants, several attempts were made to remedy the painful sensations reported by these subjects (e.g., ensuring the sponges were adequately soaked, rearranging hair to increase scalp contact, ensuring the electrodes were firmly against the scalp), but the subjects ultimately withdrew. Nevertheless, the remaining subjects that tolerated the PRE-STIM TICKLE were able to complete the 20 min of tDCS (both sham and 4 mA) and the FTs.

This study provides early evidence about the tolerability and efficacy of tDCS using a higher intensity (4 mA) than most clinical trials (≤2 mA). Investigations like this are required to expand the tDCS parameter space and help ensure optimization of the efficacy of tDCS for clinical studies. This is especially vital because, depending on the baseline excitability, different stimulation intensities may have a different maximum efficacy [45]. In other words, clinical populations that have decreased brain activity/excitability from disease or injury could potentially benefit from higher intensity (>2 mA) tDCS. Thus, systematic exploration of the tolerability and effects of different tDCS parameters is critical for the identification of maximally efficient approaches.

The fatigability results primarily indicated differences between the knee extensor and flexor muscle groups. These findings were expected based on previous understandings of knee extensor/flexor differences [46] and are similar to those found by others [47]. However, there was an increased fatigability of the left knee flexors in the 4 mA condition (Figure 3). One potential explanation is that 4 mA tDCS increased neural drive, which would alter muscle activation patterns (increased motor unit recruitment and/or discharge rate [48]) of the left knee flexors during the FT. Such altered activation patterns, particularly at the beginning of the FT, could theoretically increase fatigability by prematurely exhausting the motor units and decreasing their ability to help maintain maximal effort at the end of a task, which would subsequently increase the FI. In addition, there is some evidence that tDCS may help decrease pain [31,49]. Less effort-related muscular pain during the FT could result in increased motivation [22], improved exercise tolerance (i.e., lower rating of perceived exertion [50]), or enhanced intermuscular coherence (improved cooperation of agonist muscles [22,50]), which could also increase the FI of the left knee flexors. Because the FI used in this study is a function of torque production at the beginning and the end of the fatigue test, a greater FI must stem from (1) increase torque production at the beginning, (2) decrease torque production at the end, or (3) a combination of 1 and 2. Thus, the increased leg muscle fatigability found in the 4 mA tDCS condition of this study may have been from an increased torque production of the left knee flexors (e.g., via increased motivation, exercise tolerance, or enhanced intermuscular coherence) at the beginning of the maximal effort fatigue test. These effects would be especially beneficial in populations that suffer from reduced neural drive, increased pain, and increased fatigue, as is the case in multiple sclerosis (MS). For example, if PwMS experienced less pain, increased motivation, or improved intermuscular coherence of affected limbs, then the net effect could be an improvement in quality of life and the performance of daily activities.

Additionally, the increased fatigability of the left (non-dominant) flexors in the 4 mA condition, which was over the dominant (left) M1, is also a novel finding. This suggests that tDCS of the dominant hemisphere may influence the non-dominant hemisphere, which has also been proposed elsewhere [40,51,52]. For example, the results of Mondini et al. [51] indicated effects of tDCS on spectral EEG power on the side contralateral to stimulation and Park et al. [52] found that stimulation of the left dorsolateral prefrontal cortex had diffuse effects on right hemisphere areas. The present finding also supports the idea of tDCS affecting interhemispheric cooperation of the primary motor cortices during motor performance [53] and may also help explain the promising findings of bilateral tDCS montages [23,54]. Certainly, there are other possible explanations that require further investigation. For example, some subjects respond to tDCS, while some do not [55,56,57] and some subjects may have brain connectivity that allows the current to flow in the preferential direction [58], which may not be the case for others. Furthermore, M1 to supraorbital montages do not, in general, stimulate the motor system in a consistent way [6] and two supraorbital active electrodes may allow a great amount of current to pass through the orbit; in either of these montages, it is also possible that some structures located behind the eye (e.g., lower surface of the frontal cortex) are also affected by the stimulation. Another speculation is that a ceiling effect of the targeted motor cortex exists, which might increase transcallosal inhibition and enhance fatigability in the ipsilateral limb. Thus, more physiological tests with transcranial magnetic stimulation (TMS) and electroencephalography (EEG) are needed. Lastly, because of the lack of physiological data for a 4 mA tDCS intensity, there is also a possibility that the cathode might also be excitatory [59]. Specifically, in any brain region where current is applied there may be a combination of ‘anodal (excitatory)’ and ‘cathodal (inhibitory)’ effects depending on the orientation of specific neurons within the induced field and some neurons, e.g., with star-shaped dendrites (including inhibitory interneurons), may be equally affected by both field orientations (i.e., anodal or cathodal) [60].

A few limitations are noted for this study. First, although this study recruited more subjects (*n* = 31) than other tolerability reports ([16] *n* = 18), it was not as large as other investigations ([19] *n* = 50 young, healthy; [1] *n* = 77 young, healthy; [15] *n* = 512 young, healthy). Therefore, the relatively small number of subjects in this study may caution generalization. Additionally, leg muscle fatigability was investigated in young, neurologically healthy subjects that were at least moderately active. It is possible that such a sample may have experienced a ceiling effect of fatigability improvements, which could have blunted the tDCS effects. Thus, a sample that includes a population with fatigue as a primary symptom, like MS, may have more potential for improvement and might yield different fatigability results. Additionally, one of the early signs of MS is weakness in one limb, especially the leg, which has been determined to be a significant cause of the progressive worsening of walking abilities in PwMS [61]. Furthermore, progressive weakness of the knee flexor muscle group has been found to be a major contributor to the increased impairment in walking ability [62] and early fatigability in PwMS [63]. Thus, tDCS might have the potential to increase neuronal excitability in the affected leg of PwMS [10].

An additional limitation is that a measure of M1 excitability was not obtained, and therefore the effects of 4 mA tDCS on cortical excitability remain uncertain. Often, TMS is used to measure the magnitude and duration of excitability changes and could be used to verify and compare excitability changes in future studies. Furthermore, leg muscle activity (i.e., EMG) was not collected. As mentioned above, it is possible that greater EMG activity, especially at the beginning of the FT, of the left flexors in the 4 mA condition may help explain the present finding of increased fatigability. Also, isokinetic testing, like that in the present study, may not have applicability to functional activities of everyday life [64]. Lastly, the current study was a combination of tDCS administered before and during a task, but the best window for the application of tDCS has not been established. It has been proposed that stimulation should be administered during a task [20], but there is also evidence that the effects of tDCS may not peak for several min (e.g., 60–90 min) after the stimulation period [65].

Future studies should continue to expand upon the effects of higher intensity stimulation (>2 mA) and to compare the effects of lower intensities (≤2 mA) to higher intensities to determine the dose–response of increasing tDCS intensity. This is particularly important because there is currently incomplete evidence that increasing current intensity of tDCS improves outcomes [66]. Understanding the dose–response in human tDCS studies is required for protocol optimization, including individualized doses to reduce outcome variability and safety concerns. Furthermore, such analyses are justified because there is evidence that increasing stimulation intensity or time may reverse the effects of anodal tDCS from excitatory to inhibitory [65,66,67]. Such investigations would also benefit from including TMS and EMG to determine the changes in cortical excitability and the effects on muscle activity. Future research should also study clinical populations that have the potential for increased benefit of higher intensity stimulation (e.g., stroke, Parkinson’s disease, multiple sclerosis). Additionally, the ideal timing of tDCS (i.e., before, during, after) should be systematically investigated to optimize tDCS applications. Also, future studies would benefit from the inclusion of neuroimaging to better investigate the effects of different intensities of tDCS on brain activity. Finally, future investigations should also explore the safety of acute and long-term applications of higher intensity (>2 mA) stimulation.

## 5. Conclusions

The 4 mA tDCS condition resulted in an encouraging tolerability profile, and had effects on leg fatigability in healthy young adults. Nevertheless, given that most tDCS protocols are designed to influence clinical populations that may have decreased brain excitability, exploration of higher intensity tDCS (>2 mA) in such populations is still warranted. However, given that two subjects in this study experienced moderate—Severe headache during 4 mA tDCS, which were more severe than other tolerability reports [68], administration of higher intensity tDCS should be carefully and considerately performed, especially in headache-prone or headache-sensitive populations.

## Figures and Tables

**Table 1:** Blinding and tolerability results for sham and 4 mA tDCS (*n* = 31).

Sensation ^1^	Sham	4 mA
Blinding ^2^ Accuracy	66.7%	45.2%
Tingling	1.5 ± 1.0 (*n* = 13)	2.8 ± 1.6 (*n* = 11)
Burning	3.1 ± 1.5 (*n* = 10)	4.6 ± 1.7 (*n* = 18)
Itching	2.3 ± 1.3 (*n* = 8)	3.5 ± 2.0 (*n* = 12)
Prickling	2.8 ± 1.1 (*n* = 5)	3.0 ± 1.4 (*n* = 2)
Poking	3.5 ± 3.5 (*n* = 2)	3.0 ± 1.4 (*n* = 2)
Stinging	2.3 ± 1.5 (*n* = 3)	
Watery Eyes	1.0 ± 0.0 (*n* = 1)	2.0 ± 0.0 (*n* = 1)
Needle		3.5 ± 2.1 (*n* = 4)
Headache		7.0 ± 1.4 (*n* = 2)
Pinching		2.0 ± 0.0 (*n* = 1)
Tickling		2.0 ± 0.0 (*n* = 1)
Pressure		7.0 ± 0.0 (*n* = 1)

^1^ Sensation data were collected with a 10-point Likert-type scale, with 1 = low and 10 = high. Data are mean ± SD with the number of subjects that reported a given sensation in parentheses. ^2^ Blinding results are percent of correct guesses.
